# Apolipoprotein E genetic variation, atherogenic index and cardiovascular disease risk assessment in an African population: An analysis of HIV and malaria patients in Ghana

**DOI:** 10.1371/journal.pone.0284697

**Published:** 2023-05-03

**Authors:** Nicholas Ekow Thomford, Akwasi Anyanful, Richmond Owusu Ateko, Dee Blackhurst, Robert Peter Biney, Dennis Boadi, Samuel Badu Nyarko, Martins Ekor, George Boateng Kyei

**Affiliations:** 1 Department of Medical Biochemistry, Pharmacogenomics and Genomic Medicine Group, School of Medical Sciences, College of Health and Allied Sciences, University of Cape Coast, Cape Coast, Ghana; 2 Division of Human Genetics, Department of Pathology, Faculty of Health Sciences, University of Cape Town, Cape Town, South Africa; 3 Department of Chemical Pathology, University of Ghana Medical School University of Ghana, Legon, Accra, Ghana; 4 Division of Chemical Pathology, Department of Pathology, Faculty of Health Sciences, University of Cape Town, Cape Town, South Africa; 5 Department of Pharmacology, School of Medical Sciences, College of Health and Allied Sciences, University of Cape Coast, Cape Coast, Ghana; 6 Department of Virology, Noguchi Memorial Institute for Medical Research, University of Ghana, Legon, Ghana; 7 Department of Medicine, Washington University School of Medicine, St. Louis, MO, United States of America; The University of the West Indies, JAMAICA

## Abstract

**Background:**

Apolipoprotein E is involved in lipid transport and clearance of lipoprotein through low-density lipoprotein receptors (LDLR). ApoE variation has been linked to cardiovascular disease (CVD) risk. There are 3 isoforms of ApoE which originate from two non-synonymous single nucleotide polymorphisms denoted as ε2, ε3 and ε4. The ε2 isoform is implicated in higher levels of atherogenic lipoprotein with the ε4 isoform causing LDLR downregulation. This leads to variable effects and differential CVD risk. Malaria and HIV are life-threatening diseases affecting several countries globally especially in sub-Saharan Africa. Parasite and viral activities have been implicated in lipid dysregulation leading to dyslipidaemia. This study examined ApoE variation and CVD risk assessment in malaria and HIV patients.

**Methods:**

We compared 76 malaria-only, 33 malaria-HIV coinfected, 21-HIV-only and 31 controls from a tertiary health facility in Ghana. Fasting venous blood samples were taken for ApoE genotyping and lipid measurements. Clinical and laboratory data were collected with ApoE genotyping performed using Iplex Gold microarray and PCR-RFLP. Cardiovascular disease risk was calculated using the Framingham BMI and cholesterol risk and Qrisk3 tools.

**Results:**

The frequency of C/C genotype for rs429358 was 9.32%, whiles T/T genotype for rs7412 was found in 2.48% of all participants. ε3/ε3 was the most distributed ApoE genotype accounting for 51.55% of the total participants whiles ε2/ε2 was found in 2.48% of participants, with 1 in malaria-only and 3 in HIV-only patients. There was a significant association between ε4+ and high TG (OR = 0.20, CI; 0.05–0.73; p = 0.015), whiles ε2+ was significantly associated with higher BMI (OR; 0.24, CI; 0.06–0.87; p = 0.030) and higher Castelli Risk Index II in females (OR = 11.26, CI; 1.37–92.30; p = 0.024). A higher proportion of malaria-only participants had a moderate to high 10-year CVD risk.

**Conclusion:**

Overall malaria patients seem to have a higher CVD risk though the means through which this occurs may be poorly understood. ε2/ε2 genotypes was observed in our population at a lower frequency. Further studies are vital to determine CVD risk in malaria and how this occurs.

## Introduction

Apolipoprotein E is a lipid transport protein and an important ligand for low-density lipoprotein (LDL) receptors with a function in cholesterol metabolism and cardiovascular diseases (CVD) [1, 2]. Apolipoprotein (Apo) E genes are involved in lipoprotein synthesis and several metabolic processes, and their dysregulation has become a significant link in understanding susceptibility and risks in cardiovascular diseases (CVD) [3, 4]. Globally, CVD represents one of the leading health challenges [5, 6]. ApoE has a more powerful role in the clearance of (remnant) lipoproteins through the low-density lipoprotein receptor (LDLR) (as well as some related receptors) and significantly also, heparan sulphate proteoglycans [7, 8]. In addition, ApoE genotypes have been implicated in the modification of response to polyunsaturated fatty acids through control of enzyme expression and methylation [9–11].

Due to the vital role of ApoE in the transport and metabolism of lipids, several questions have arisen about ApoE genotypes and how they modulate fatty acids in CVD. Three variants of ApoE are encoded by a gene on chromosome 19q13.2. Three primary isoforms/variants of this gene originate from two non‐synonymous single nucleotide polymorphisms (SNPs) (rs429358 and rs7412), referred to as ε2, ε3 and ε4. These three common alleles ε2, ε3 and ε4 constitute polymorphisms found in most populations resulting in six (6) genotypes ε2/ε2, ε3/ε3, ε4/ε4, ε2/ε4, ε2/ε3 and ε3/ε4. The population distribution of ApoE alleles and genotypes shows the ε3 variant and ε3ε3 genotypes as the most commonly occurring in all studied populations and considered the wild type, whiles the ε2ε2 is least represented [12–14]. The ε2 and ε4 alleles have been implicated in cardiovascular diseases. The ε2 allele increases atherogenic lipoprotein levels through poor binding to LDL receptors (LDLRs), whilst ε4 increases LDLR downregulation [6].

Both malaria and HIV potentially cause lipid dysregulation, and the variable distribution of ApoE alleles and genotypes among different populations may predispose individuals to CVD risk. Malaria and HIV are life-threatening diseases affecting more than 100 countries globally, especially in sub-Saharan African (SSA) countries. Malaria involves a complex maze of vertebrate host-parasite interactions that affect both the host and parasite. The parasite’s survival relies on vertebrate host metabolic processes via metabolite exchange to ensure its survival and proliferation [15, 16]. The causative organism of malaria, *Plasmodium falciparum*, has a liver stage where sporozoites invade hepatocytes which causes organ congestion, sinusoidal blockage, and cellular inflammation. The liver serves as a central metabolic organ in glucose and lipid metabolism regulation through gluconeogenesis, β-oxidation, lipogenesis and uptake and secretion of lipoproteins [17, 18]. For the P. *falciparum* to make it through its lifecycle in the host, they manipulate the host’s lipid metabolic pathways since they cannot synthesize lipid classes that are fundamental for their development and replication [19]. P. *falciparum*, therefore, causes lipid dysregulation as the parasite uses cholesterol and phospholipids from its host to increase the surface area and volume of its internal membranes [20–23].

In HIV, dyslipidemia presents with distinct patterns. In patients who are not receiving antiretroviral therapy (ART) for their infection, high-density lipoprotein cholesterol (HDL-C), total cholesterol (TC) and low-density lipoprotein cholesterol (LDL-C) decrease while triglyceride (TG) increases However, after ART initiation, TC and LDL-C increase while HDL-C remains low [24–26]. This study examined ApoE variants and CVD risk assessment in malaria and HIV patients attending a tertiary health facility in Cape Coast, Ghana, to understand how ApoE variation influences CVD risk in this cohort.

## Materials and methods

### Study subjects

The research was conducted according to the code of ethics of the Helsinki declaration. Ethical clearance was obtained from the Cape Coast Teaching Hospital Ethical Review Committee (CCTHERC/EC/2020/2020/109). Written or verbal informed consent was obtained from each participant or legal guardian. Patients were recruited from the outpatient departments of Cape Coast Teaching Hospital, Ewim Polyclinic, Cape Coast Metropolitan Hospital and Moree Health Post, all in the Central Region of Ghana. Participants in this study were either malaria patients, HIV patients, malaria-HIV patients or controls with high lipid profiles. We used individuals with high lipid profiles as controls because there is no data in this population and for comparative purposes of proportions in malaria-only, HIV-only and malaria-HIV cohorts. Data on age, gender, employment, ethnicity, education and smoking status were collected using a structured questionnaire using a computerized assisted personal interview (CAPI) tool, KoboToolbox [27]. Height and weight were measured using a stadiometer and a digital scale and used to compute the body mass index (BMI) of each participant.

### Blood sampling, atherogenic indices, lipid ratio evaluation and laboratory analysis

Blood samples and relevant clinical and medical history were collected on the day of recruitment. Whole blood was collected into ethylenediamine tetraacetic acid (EDTA) vacutainer tubes for DNA extraction, and the plasma was separated for lipid profile analysis. Biochemical tests involving lipid profile on total cholesterol (TC), triglycerides (TG) and High-Density Lipoprotein-cholesterol (HDL-C) was analysed using Selectra Pro XL autoanalyzer (ElitechGroup, Puteaux, France). Non-HDL-C, LDL-C and TC/HDL-C ratios were then estimated. Atherogenic ratios and indices were calculated as follows according to [28, 29]

$$Atherogenic\;Index\;of\;Plasma\;(AIP)=\frac{log\;TG}{HDL-C}$$

$$Castelli’s\;Risk\;Index\;(CRI-I)=\frac{TC}{HDL-C}$$

$$Castelli’s\;Risk\;Index\;(CRI-II)=\frac{LDL-C}{HDL-C\;}$$

$$Atherogenic\;Coefficient\;(AC)=\frac{\left(TC–HDL-C\right)}{HDL-C\;}$$

### DNA extraction and APOE genotype

DNA was extracted from the previously collected whole blood of each participant using E.Z.N.A^®^ blood DNA mini kit (Omega Bio-tek, Inc. Norcross, USA) according to the manufacturer’s instructions. Extracted DNA was diluted to a minimum concentration of 20ng/uL for genotyping procedures. Genotyping of rs7412 and rs429358 polymorphisms on ApoE were undertaken using Iplex GOLD SNP genotyping protocol on the Agena MassARRAY^®^ system (Agena Bioscience^TM^, San Diego, CA, USA) and polymerase chain reaction restriction fragment length polymorphism (PCR-RFLP) (F1-GGCACGGCTGTCCAAGGA; R-CTCGCGGATGGCGCTGAG, Enzyme HhaI). Products after restriction digestion were viewed on 3% agarose gel and ApoE genotype was determined according to the bands obtained. The rs7412 and rs429358 genotype combinations were used to make the call for ApoE genotype with confirmation undertaken for randomly selected samples (Table 1).

**Table 1:** ApoE genotypes using rs429358 & rs7412.

rs429358	rs7412	rs429358 & rs7412ApoE genotype
**C/T**	C/T	ε2/ε4
**T/T**	T/T	ε2/ε2
**T/T**	C/T	ε2/ε3
**T/T**	C/C	ε3/ε3
**C/T**	C/C	ε3/ε4
**C/C**	C/C	ε4/ε4

### Cardiovascular risk estimation

Ten-year cardiovascular risk was assessed by calculating the Framingham risk score (FRS) and Qrisk3 using the validated Framingham BMI risk, Framingham cholesterol risk and Qrisk3 tools for estimating CVD in our cohort [30–32] at the time of study enrolment. Each risk score tool has age limits, and therefore those that fell out of the range of the age limits were excluded.

### Data analysis

Data obtained are presented as numbers with frequencies and percentages for categorical variables. Biochemical parameters are presented as means with standard deviations and medians with interquartile ranges in box and violin plots. Due to the variable functions of the different isoforms of ApoE in lipid metabolism [33, 34], analysis was undertaken to factor in the type of ApoE allele an individual carries (ε2+ carriers, ε3/ε3 homozygous reference and ε4+ carriers) and the ApoE genotypes which gave rise to six (6) genotypes (i) ε2/ε2 (ii) ε3/ε3 (iii) ε4/ε4 (iv) ε2/ε4 (v) ε2/ε3 (vi) ε3/ε4. ApoE carrier status was undertaken by combining ε2/ε2 and ε2/ε3 as ε2+ while ε4/ε4 and ε3/ε4 were grouped as ε4+ carriers. Univariate and multivariate logistic regression analyses were performed to find association of elevated lipid parameters contributing to CVD. Distribution of lipid in comparison with ApoE genotypes and carrier status were presented as box and violin plots with data represented as median and interquartile ranges and Mann-Whitney U test for comparing the various groups. Kruskall-wallis test was used to test for multiple comparisons. All statistical analyses, graphs and calculations were performed using STATA, version 17 (StataCorp, College Station, Texas, USA), excel 2019 and GraphPad Prism 9 for Mac (GraphPad Software, San Diego, CA, USA).

## Results

### Clinicodemographic data of participants

The mean age of the participants was 37 ± 16 years. Seventy-one (71%) percent of our participants were females with a mean age of 39 ± 16 years. Most participants were between 20–59 years. Concerning BMI, 28 individuals (17%) were overweight, and 29 individuals (18%) were obese. Over 96% of the participants were non-smokers, while 26.25 regularly used alcohol. Using at least one NCEP-ATP III criterion based on low HDL, high TG, high HDL and high TC, 17.10% and 15.79% of malaria-diagnosed patients had high TG and low HDL, respectively. Table 2 summarizes the clinicodemographic data of the participants. The medications that were used to treat malaria, HIV and manage dyslipidaemia were artemether-lumefantrine, dolutegravir-tenofovir-lamivudine (DTG/TFD/3TC) and statins.

**Table 2:** Clinicodemographic data of participants.

Variables	Malaria	HIV	Malaria-HIV	CONTROLS	p-value
	(n = 76)	(n = 33)	(n = 21)	(n = 31)	
Male	27(35.53)	3(9.09)	5(23.81)	11(35.81)	0.831
Female	49(64.47)	30(90.91)	16(76.19)	19(76.19)	
**Age**					
0–19	19 (25)	0(0)	3(14.29)	0(0)	0.656
20–29	12 (15.59)	7(21.21)	1(4.76)	8(25.81)	
30–39	11 (14.47)	9(27.27)	3(14.29)	7(22.58)	
40–49	10 (13.16)	9(27.27)	9(42.86)	7(22.58)	
50–59	14 (18.42)	6(18.18)	5(23.81)	3(9.68)	
60–69	7(9.21)	1(3.03)	0(0)	2(6.45)	
70–79	3(3.95)	0(0)	0(0)	2(6.45)	
**BMI**					
0–18.49	11(14.47)	3(9.09)	3(14.29)	5(16.13)	0.582
18.5–24.9	42(55.26)	17(51.52)	12(57.14)	11(35.48)	
25.29.9	11(14.47)	6(18.18)	4(19.05)	7(22.58)	
>30	12(15.79)	7(21.21)	2(9.52)	8(25.81)	
**Smoking**					
Never used	75(98.68)	31(93.94)	21(100)	30(96.77)	0.913
Irregularly used	1(1.32)	1(3.03)	0(0)	1(3.23)	
Currently using	0(0)	1(3.03)	0(0)	0(0)	
**Alcohol use**					
Regularly use	4(5.26)	0(0)	1(4.76)	1(3.23)	0.881
Irregularly use	16(21.05)	7(21.21)	7(33.33)	6(19.35)	
Never used	56(73.68)	26{78.79)	13(61.90)	24(77.42)	
**SBP (mmHg)**					
1–120	51(67.11)	22(66.67)	13(61.90)	19(61.29)	0.415
121–139	12(15.79)	5(15.15)	5(23.81)	8(25.81)	
>140	13(17.11)	6(18.18)	3(14.29)	4(12.90)	
**DBP (mmHg)**					
1–80	51(67.11)	22(66.67)	14(66.67)	24(77.41)	0.606
81–89	9(11.84)	3(9.09)	3(14.29)	3(9.68)	
>90	16(21.05)	8(24.24)	4(19.05)	4(12.90)	
**Ethnicity**					
Akan	71(93.42)	33(100)	21(100)	21(67.74)	0.147
Ewe	3(3.95)	0(0)	0(0)	2(6.45)	
Frafra	0(0)	0(0)	0(0)	2(6.45)	
Dagbani	1(1.32)	0(0)	0(0)	1(3.23)	
Akuapem-Larteh	1(1.32)	0(0)	0(0)	0(0)	
**Highest Level of Education**					
Primary	25(32.89)	6(18.18)	9(42.86)	3(9.68)	0.000*
JHS	23(30.26)	10(30.30)	5(23.81)	2(6.45)	
SHS	10(13.16)	4(12.12)	1(4.76)	6(19.35)	
Tertiary	3(3.95)	2(6.06)	1(4.76)	14(45.16)	
No formal education	15(19.74)	9(27.27)	5(23.81)	3(9.68)	
**Lipid Profile (**mmol/L)					
TC	3.87± 1.39	4.08 ± 1.06	3.25 ± 1.11	5.57 ± 1.40	0.053
TG	1.30 ± 0.71	1.39 ± 0.81	1.49 ± 1.13	1.62 ± 1.17	0.263
HDL-C	1.12 ± 0.69	1.06 ± 0.47	1.35 ± 1.09	1.62 ± 0.75	0.068
LDL-C	2.112 ± 1.22	2.38 ± 0.83	1.22 ± 1.79	3.30 ± 1.03	0.020*
Non-HDL-C	2.74 ± 1.25	3.02 ± 0.85	4.77 ± 3.84	4.08 ± 1.46	0.247
Chol/HDL ratio	4.34 ± 2.77	4.48 ± 2.50	4.03 ± 2.77	3.88 ± 1.48	0.349
**Dyslipidaemia indices**					
High TC	3 (3.94)	1 (3.03)	0 (0.00)	10 (32.25)	0.001*
High TG	13 (17.10)	5 (15.15)	4(19.05)	10 (32.25)	0.165
High LDL-C	4 (5.26)	2 (6.06)	0 (0.00)	18 (58.06)	0.001*
Low HDL-C	12 (15.79)	3 (9.09)	1 (3.33)	10 (32.25)	0.338

Dyslipidaemia is defined as the presence of at least one NCEP-ATP III criterion using the following parameters low HDL-C (<1.03 mmol/L in males; <1.29 mmol/L in females), high TG (≥1.7 mmol/L, TC >6.2 mmol/L and LDL-C >3.37 mmol/L), p*<0*.*05* is considered statistically significant

### APOE genotypes

Of our total participants, 15 exhibited the C/C genotype for rs429358, accounting for 9.32%. Only 4 subjects were identified with T/T genotype for rs7412, accounting for 2.48% of all participants. Among the various disease categories, C/C genotype distribution was 7.84% among malaria-only patients, 6.06% among HIV-only patients, 9.92% among malaria-HIV co-infected patients and 16.13% among dyslipidaemia controls (Table 3). The distribution of rs7412 genotypes showed only 4 subjects with T/T genotypes, with 1 observed in malaria-only and 3 in HIV-only patients. ε3/ε3 was the most distributed ApoE genotype accounting for 51.55% of the total participants. ε2/ε2 was found in 2.48% of participants, with 1 in malaria-only and 3 in HIV-only patients. ApoE3 was the most frequently distributed allele (51.55%), followed by ApoE2 (24.22%) and ApoE4 (8.70).

**Table 3:** Genotype distribution in various groups.

Variables	Malaria	HIV	Malaria-HIV	CONTROLS	p-value
	(n = 76)	(n = 33)	(n = 21)	(n = 31)	
***APOE rs429358* (T>C)**					
T/T	59(77.63)	21(63.64)	15(71.43)	19(61.29)	0.392
C/T	11(14.47)	10(30.30)	4(19.05)	7(22.58)	
C/C	6(7.84)	2(6.06)	2(9.52)	5(16.13)	
***APOE rs7412* (C>T)**					
C/C	55(72.37)	19(57.58)	19(90.48)	30(96.77)	0.002*
C/T	20(26.32)	11(33.33)	2(9.52)	1(3.23)	
T/T	1(1.32)	3(9.09)	0(0)	0(0)	
**APOE Genotype frequency**	**Malaria**	**HIV**	**Malaria-HIV**	**CONTROLS**	
	**(n = 76)**	**(n = 33)**	**(n = 21)**	**(n = 31)**	
ε2/ε4	3(3.95)	4(12.12)	0(0)	0(0)	0.017*
ε3/ε3	41(53.95)	11(33.33)	13(61.90)	18(58.06)	
ε2/ε3	18(23.68)	7(21.21)	2(9.52)	1(3.23)	
ε3/ε4	8(10.53)	6(18.18)	4(19.05)	7(22.58)	
ε4/ε4	5(6.58)	2(6.06)	2(9.52)	5(16.13)	
ε2/ε2	1(1.32)	3(9.09)	0(0)	0(0)	
**Allele frequency**					
ε2	22(28.95)	14(18.42)	2(9.52)	1(3.23)	0.006*
ε3	49(64.47)	17(51.52)	17(80.95)	25(80.63)	
ε4	5(6.58)	2(6.06)	2(9.52)	5(16.13)	
**ApoE carrier status**					
ε2+	19(25)	10(30.30)	2(9.52)	1(3.23)	0.012*
ε3/ε3	41(53.95)	11(33.33)	13(61.90)	18(58.06)	
ε4+	13(17.11)	8(10.53)	6(28.57)	12(57.14)	

Analysis of APOE *rs429358* shows significant differences in the distribution of TG and HDL-C among the T/T, C/T and C/C genotypes (Table 4). C/C homozygous mutants had a mean TG of 1.8 ± 1.64 mmol/L. The *APOE rs429358* genotypes had non-significant differences among the various lipid parameters.

**Table 4:** Serum lipid parameters according to ApoE variants.

Variables	*APOE rs429358*			p-value	*APOE rs7412*			p-value
(mmol/L)								
	T/T	C/T	C/C		C/C	C/T	T/T	
TC	4.05 ± 1.43	4.7 ± 1.7	4.7 ± 1.34	0.305	4.42 ± 1.56	3.78 ± 1.27	3.45 ± 0.83	0.495
TG	1.29 ± 0.79	1.58 ± 0.71	1.8 ± 1.64	0.043*	1.47 ± 0.94	1.15 ± 0.65	2.04 ± 1.56	0.567
HDL-C	1.27 ± 0.73	1.20 ± 0.57	1.30 ± 1.15	0.027*	1.29 ± 0.81	1.17 ± 0.51	0.87 ± 0.49	0.523
LDL-C	2.17 ± 1.28	2.90 ± 1.50	2.60 ± 0.89	0.754	2.47 ± 1.41	2.07 ± 1.02	1.65 ± 0.38	0.761
Non-HDL-C	3.11 ± 1.85	3.87 ± 1.66	3.41 ± 1.30	0.092	3.53 ± 1.94	2.62 ± 0.96	2.58 ± 0.33	0.972
Chol/HDL ratio	4.08 ± 2.67	4.43 ± 1.73	4.89 ± 1.96	1.000	4.34 ± 2.47	3.83 ± 2.41	4.40 ± 1.55	1.000

**Fig 1:**
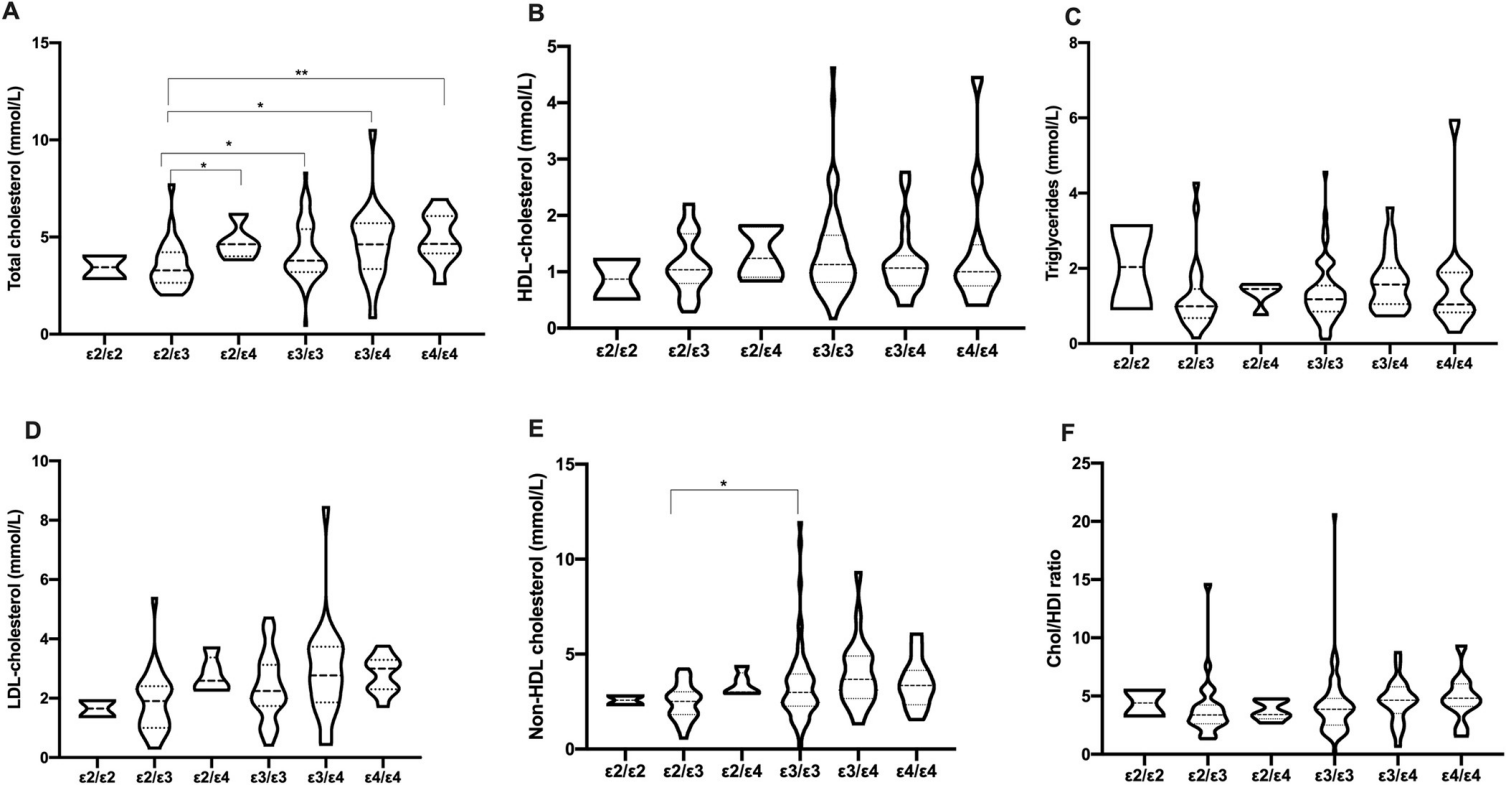
Distribution of lipids parameters according to ApoE genotypes.

As shown in Fig 1 there were no significant differences in the lipid parameters distributed among the ApoE genotypes. However, ε2/ε2 genotypes had the highest median TG of 2.04 (0.93–3.14) mmol/L, which is above the upper limit of reference and the lowest HDL of 0.87 (0.52–1.22) mmol/L. There were no significant differences between most of the genotypes and lipid distribution. However, there was significance in TC between ε2/ε3 and ε2/ε4, ε3/ε3, ε3/ε4, ε4/ε4 (Fig 1A). There were significant differences in non-HDL cholesterol between ε2/ε3 and ε3/ε3 (Fig 1E).

### Distribution of abnormal atherogenic indices in participants

Across the various stratified disease groups, atherogenic indices were compared (Table 5). Using the indices, HIV-only patients had the highest proportion of abnormal atherogenic indices, putting them at risk of CVD. ApoE ε4+ allele carriers seem to have high proportions of abnormal atherogenic indices in comparison to ε3/ε3 (Table 6).

**Table 5:** Distribution of abnormal atherogenic indices among the study population stratified by diseases.

Indices	Total (%)	Malaria	HIV	Malaria-HIV	CONTROLS	P value
		(n = 76)	(n = 33)	(n = 21)	(n = 31)	
**AIP**	58	22 (28.95)	17 (51.51)	7 (33.33)	12 (38.71)	0.863
**CRI**						
I (M)	20	10 (13.16)	2 (6.06)	3 (14.29)	5 (16.13)	0.764
I (F)	67	28 (36.84)	23 (69.96)	7 (33.33)	9 (29.03)	0.313
II	27	9 (11.84)	9 (27.27)	3 (14.29)	6 (19.35)	0.931
**AC**	64	27 (35.52)	16 (48.48)	9 (42.86)	12 (38.71)	0.814

AIP = Atherogenic index of plasma, CRI = Castelli’s risk index, AC = Atherogenic coefficient. The following are the abnormal values of AIP, lipid ratios, and CHOLIndex for cardiovascular risk: AIP >0.1, CRI-I >3.5 in males and >3.0 in females, CRI-II >3.3, AC >3.0 [28, 35, 36]

**Table 6:** Distribution of abnormal atherogenic indices among ApoE allele carrier status.

Indices	*ε3/ε3* (n = 83)	*ε2+* (n = 32)	*ε4+* (n = 39)	*P value*
**AIP**	29 (34.94)	14 (43.75)	13 (33.33)	0.449
**CRI**				
I (M)	12 (41.38)	2 (28.57)	6 (75.00)	0.084
I (F)	27 (50.00)	19 (76.00)	17 (56.67)	0.316
II	16 (19.28)	4 (12.50)	6 (15.38)	0.861
**AC**	30 (36.15)	12 (37.50)	19 (48.72)	0.790

**Fig 2:**
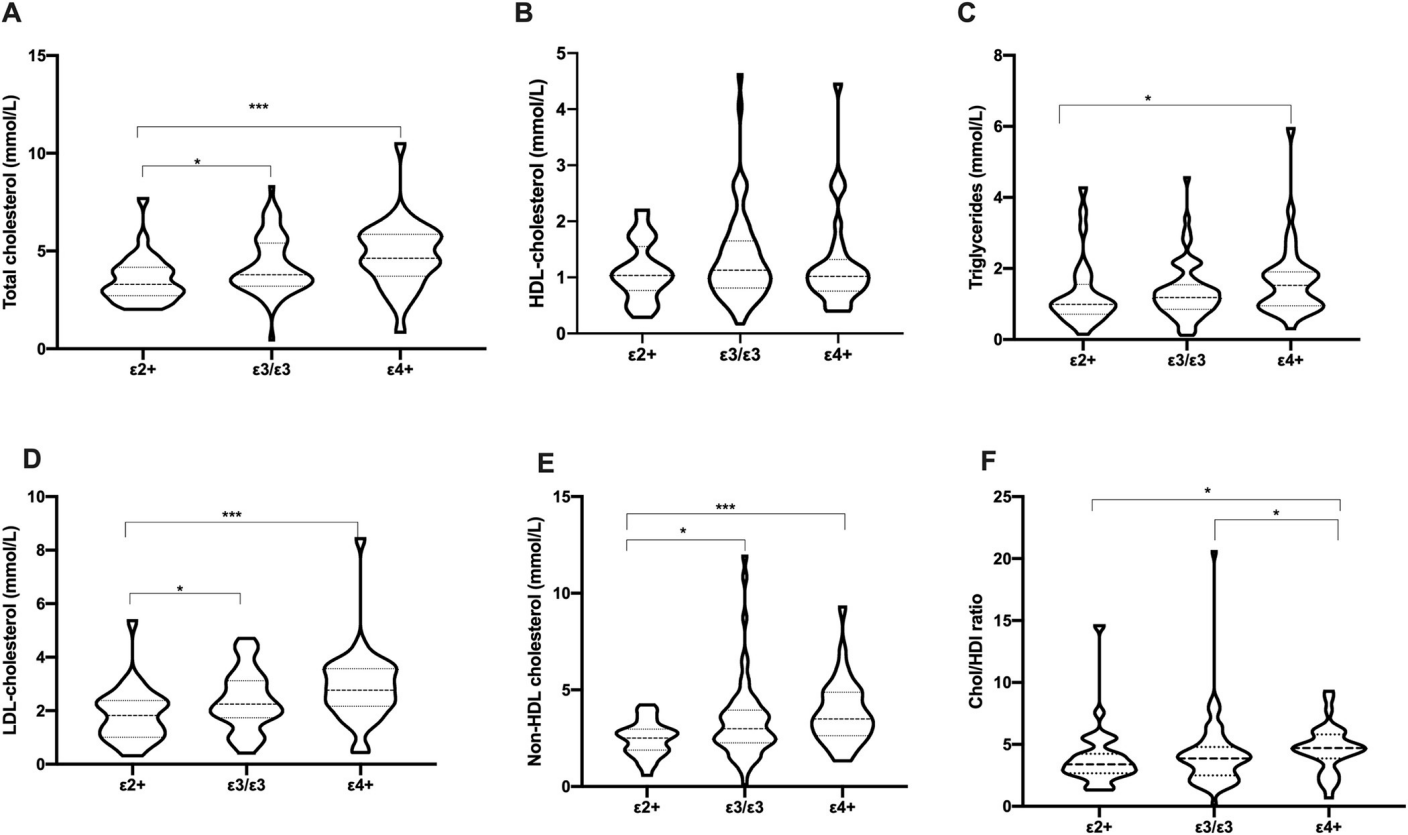
Distribution of lipids profile according to ApoE carrier status.

Significant differences were observed among the combined effects of ApoE allele carrier status and serum lipid levels in the total participants (Fig 2). *ε4+* carriers had the highest TC, LDL-C and Chol/HDL-C ratio of 4.65 (3.72–5.89), 2.77 (2.16–3.57) mmol/L and 4.71 (3.88–5.51) respectively which were near optimal.

### Comparison of ApoE carrier status and atherogenic risk indices among patient categories

The distribution of ApoE allele carrier status among participants is presented in S1 Table. There were no significant differences between the various serum lipid parameters and ApoE allele carrier status except for LDL-C in malaria-HIV co-infected and non-HDL-C in malaria-only participants. However, ε4+ allele carriers had the lowest combined HDL-C levels (0.92 ± 0.62 mmol/L).

### Analysis of atherogenic risk indices and ApoE genotypes and variants

Further analyses of a possible association of atherogenic and serum lipid indices with ApoE allele carrier status and risk alleles showed a significant association between high TG and ε4+ (OR = 0.20, CI; 0.05–0.73; p = 0.015). Carrying a risk allele for rs429358 CT/CC was found to be significantly associated (OR = 0.28, CI; 0.09–0.85; p = 0.025) with high TG levels, LDL-C (OR = 0.35 95% CI 0.13–0.86; p = 0.023), abnormal AC (OR = 3.04, CI; 1.16–7.90; p = 0.023) and abnormal AIP (OR = 3.04 CI;1.16–7.90; p = 0.023) (Table 7). ε2+ was significantly associated with higher BMI classified as overweight or obese (OR; 0.24, CI; 0.06–0.87; p = 0.030) and higher Castelli Risk Index II in females (OR = 11.26, CI; 1.37–92.30; p = 0.024) with rs7412 CT/CC genotypes being significantly associated with high BMI (OR = 0.39, CI;0.15–0.96; p = 0.041).

**Table 7:** Univariate and multivariate analysis of atherogenic risk indices and ApoE genotypes and variants.

	ε3/ε3	ApoE carrier				APOE rs429358		APOE rs7412	
		ε2+		ε4+		CT/ CC		CT/TT	
	(reference)	OR (CI)	p-value	OR (CI)	p-value	OR (CI)	p-value	OR (CI)	p-value
**TC > 6.2 mmol/L**									
Univariate		3.38 (0.40–28.83)	0.266	0.70 (0.20–2.41)	0.571	0.54 (0.17–1.67)	0.282	2.12 (0.45–10.01)	0.344
Multivariate		0.67 (0.03–13.30)	0.790	0.64 (0.09–4.58)	0.654	0.50 (0.10–2.53)	0.399	0.43 (0.05–4.06)	0.464
**TG ≥1.7 mmol/L**									
Univariate		0.95 (0.32–2.83)	0.931	0.40 (0.16–1.01)	0.053	0.45 (0.19–1.03)	0.058	2.08 (0.72–5.97)	0.172
Multivariate		0.74 (0.15–3.72)	0.711	**0.20 (0.05–0.73)**	0.015*	**0.28 (0.09–0.85)**	0.025*	3.09 (0.58–16.17)	0.182
**LDL-C >3.37 mmol/L**									
Univariate		5.71 (0.70–46.61)	0.104	0.42 (0.16–1.12)	0.083	**0.35 (0.13–0.86)**	0.023*	4.4 (0.97–19.88)	0.054
Multivariate		2.78 (0.13–56.83)	0.507	0.39 (0.08–1.82)	0.232	0.31 (0.08–1.25)	0.100	1.58 (0.19–13.38)	0.675
**HDL-C <1.03 mmol/L in males**									
Univariate		0.84 (0.14–5.07)	0.855	0.84 (0.14–5.07)	0.855	0.88 (0.18–4.17)	0.867	0.88 (0.18–4.17)	0.867
Multivariate		0.13 (00–4.72)	0.269	0.31 (00–15.72)	0.562	1.53 (0.09–25.99)	0.768	0.43 (0.02–6.53)	0.547
**HDL-C; <1.29 mmol/L in females**									
Univariate		2.06 (0.66–6.39)	0.211	2.10 (0.72–6.13)	0.176	1.71 (0.65–4.47)	0.275	1.39 (0.50–3.82)	0.526
Multivariate		0.84 (0.14–5.03)	0.849	3.77 (0.66–21.41)	0.134	2.67 (0.64–11.11)	0.177	0.35 (0.07–1.69)	0.190
**BMI >25kg/m** ^**2**^									
Univariate		0.29 (0.09–0.91)	0.035	1.58 (0.73–3.44)	0.249	1.83 (0.90–3.72)	0.093	**0.39 (0.15–0.96)**	0.041*
Multivariate		**0.24 (0.06–0.87)**	0.030*	0.92 (0.38–2.23)	0.853	1.15 (0.51–2.55)	0.740	0.42 (0.14–1.21)	0.108
**AIP>0.1**									
Univariate		0.49 (0.19–1.29)	0.149	1.29 (0.56–2.99)	0.550	1.46 (0.68–3.15)	0.330	0.48 (0.20–1.14)	0.097
Multivariate		0.47 (0.13–1.82)	0.279	2.50 (0.89–6.98)	0.081	**3.04 (1.16–7.90)**	0.023*	0.43 (0.14–1.37)	0.154
**CRI-1 >3.0 males**									
Univariate		2.00 (0.16–25.11)	0.591	6 (0.62–57.68)	0.121	5.57 (0.59–52.73)	0.134	1.44 (0.12–17.67)	0.773
Multivariate		1.40 (0.03–79.03)	0.870	4.2 (0.05–299.42)	0.508	4.13 (0.06–305.14)	0.519	1.22 (0.02–90.83)	0.928
**CRI-I >3.0 females**									
Univariate		**11.26 (1.37–92.30)**	0.024*	1.00 (0.37–2.73)	0.988	0.78 (0.30–1.98)	0.596	**13.59 (1.73–106.63)**	0.013*
Multivariate		10.82 (0.86–136.78)	0.066	0.89 (0.23–3.43)	0.864	0.75 (0.21–2.61)	0.6540	**14.12 (1.27–156.89)**	0.031*
**CRI-II>3.0**									
Univariate		0.61 (0.18–2.08)	0.438	0.61 (0.22–1.72)	0.348	0.71 (0.27–1.86)	0.497	0.78 (0.27–2.29)	0.652
Multivariate		1.56 (0.36–6.67)	0.552	0.82 (0.25–2.73)	0.745	0.71 (0.24–2.08)	0.531	1.34 (0.37–4.77)	0.654
**AC>3.0**									
Univariate		1.35 (0.52–3.48)	0.540	1.46 (0.64–3.33)	0.362	1.48 (0.69–3.15)	0.311	1.35 (0.57–3.17)	0.488
Multivariate		0.70 (0.19–2.54)	0.585	1.20 (0.44–3.27)	0.725	1.53 (0.61–3.79)	0.363	0.85 (0.27–2.63)	0.777

**atherogenic risk indices** are categorised using at least one NCEP-ATP III criteria using the following parameters low HDL-C (<1.03 mmol/L in males; <1.29 mmol/L in females), high (TG ≥1.7 mmol/L, TC >6.2 mmol/L and LDL-C >3.37 mmol/L), BMI >25kg/m^2^, p*<0*.*05* is considered statistically significant

### Cardiovascular risk assessment

**Fig 3:**
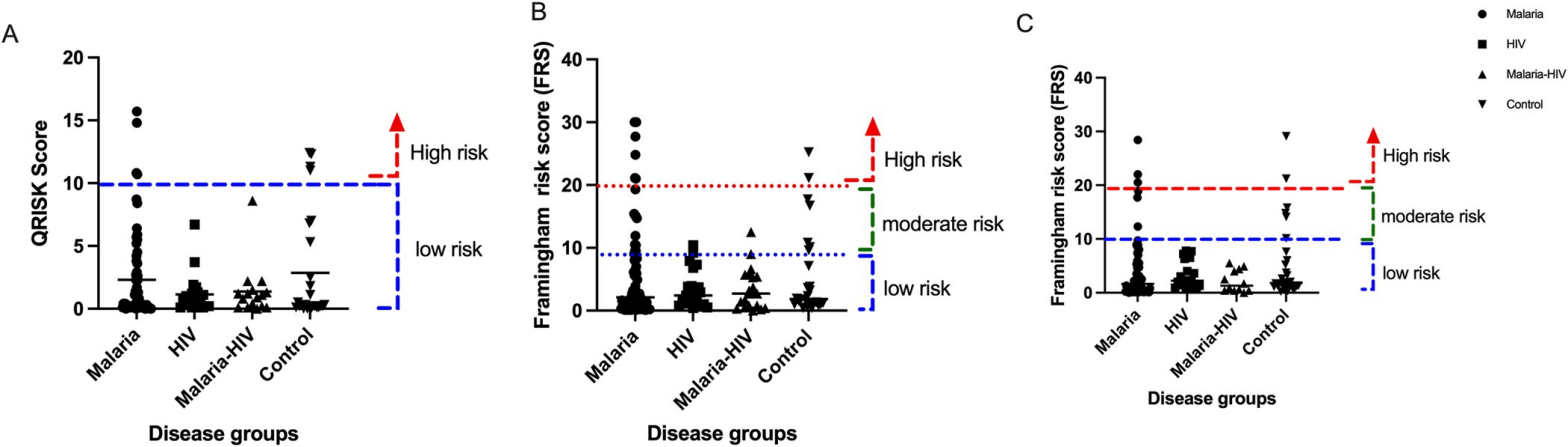
Ten-year cardiovascular risk assessment of participants stratified by groups. (A) QRISK3 calculator. (B) Framingham BMI risk calculator. (C) Framingham Cholesterol risk calculator.

Cardiovascular risk assessment was undertaken with three predictive calculators for 10-year risk (Fig 3) i.e., QRISK3 Framingham BMI risk Framingham Cholesterol. It was observed that a higher proportion of malaria-only participants had a moderate to high 10-year CVD risk. Overall, according to QRISK-3 assessment, 6.70% of malaria-only participants had an elevated CVD risk (Fig 3A). The Framingham BMI risk assessment placed 20.8% of the malaria only participants at moderate to high CVD risk (Fig 3B), whilst Framingham Cholesterol risk calculator accounted for only 9.52% of the malaria only participants at moderate to high CVD risk (Fig 3C).

**Fig 4:**
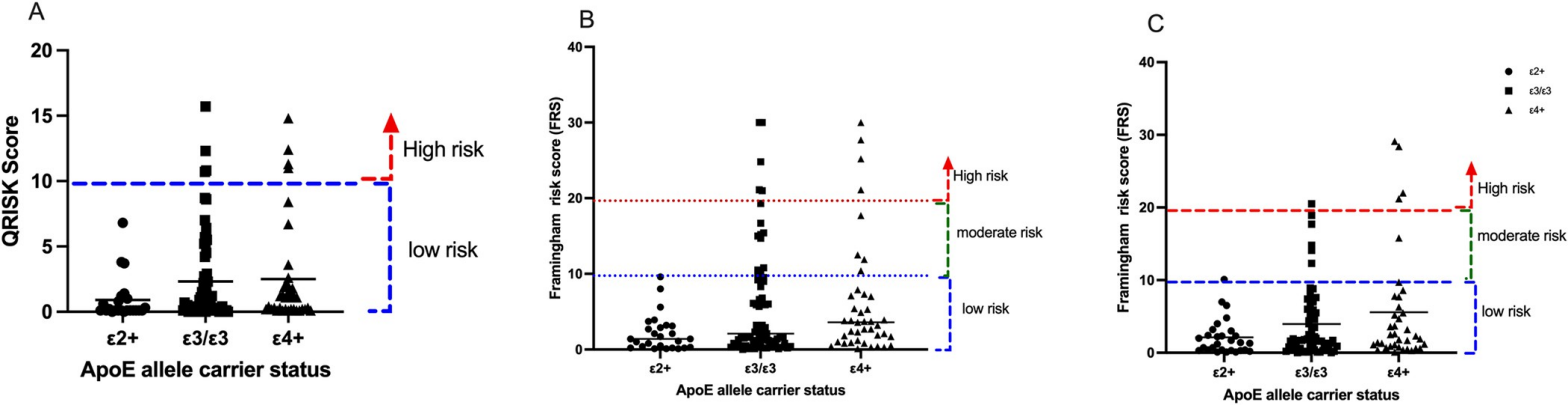
Ten-year cardiovascular risk assessment stratified by ApoE carrier status. (A) QRISK3 calculator. (B) Framingham BMI risk calculator. (C) Framingham Cholesterol risk calculator.

**Fig 5:**
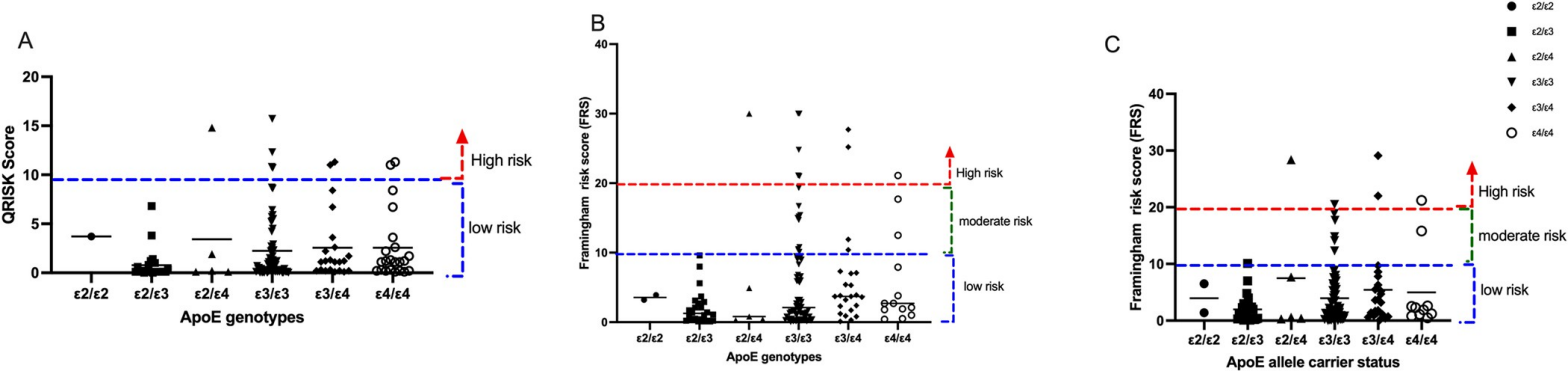
Ten-year cardiovascular risk assessment stratified by ApoE genotypes. (A) QRISK3 calculator. (B) Framingham BMI risk calculator. (C) Framingham Cholesterol risk calculator.

Overall, the ApoE allele carrier status showed that ε4+ carriers were at elevated cardiovascular risk using all three CVD estimators (Fig 4). ε4+ carrier elevated CVD risk was between 9.30–19.44% across QRISK-3, Framingham BMI and Framingham cholesterol calculators. This is further observed when the ApoE genotypes are stratified across the various assessment tools (Fig 5). It is observed that a combined higher proportion of ε3/ε4 and ε4/ε4 genotypes were at elevated CVD risks.

## Discussion

ApoE allele association with CVD has been assessed in several populations [4, 5, 37, 38]. Proposals have been made that ApoE alleles or genotypes influence lipid metabolism differences with corresponding pathologic effects [33, 39, 40, 41]. The differences in the modulation of lipid profile depending on the ApoE isoform is largely influenced by the ApoE alleles, which can be influenced by ethnicity. This study explored the associations between ApoE, atherogenic index and cardiovascular risk.

Elevated serum TC, TG and LDL-C levels were observed in malaria-only, HIV-only and malaria-HIV co-infected participants. The highest abnormal serum lipid parameters were observed in malaria patients. A metaanalysis has shown that there are observed serum lipid profile changes characteristic of malaria [23], with other studies showing elevated levels of total cholesterol, low-density lipoproteins and triglycerides in malaria-infected patients compared to controls [42–44]. A higher proportion of malaria-only participants had higher dyslipidaemia indices compared to HIV-only, malaria-HIV co-infected, and control participants, confirmed by several analyses which has shown congruent serum lipid profile changes during malaria infection [42, 45–47]. Though an explicit association between serum lipid levels and malaria pathogenesis is still in its infancy, most of the plausible hypotheses of biological mechanisms involve host lipid modifications by the parasite [19]. Abnormal serum lipid levels have previously been established in HIV patients [48–50], and in this study, we observed that mean TC, LDL-C and Chol/HDL-C ratios were high compared to malaria-only and malaria-HIV co-infected participants. Lipid dysregulation in HIV patients is understood to arise from viral modulation, uncontrolled HIV disease and the mechanism of action of ARTs [51–53].

The distribution of ApoE genotypes and alleles shows that ε2/ε2 (2.48%) genotypes were least represented in our study population, which agrees with observations in other populations [54, 55]. The most frequently occurring genotype observed was the ε3/ε3 (51.55%) followed by ε2/ε3 (17.39%), ε3/ε4 (15.53%), ε4/ε4 (8.70%) and ε2/ε4 (4.35%). These observed frequencies vary when compared to what is seen in other populations [54, 56, 57]. ApoE*ε4 allele was also least represented in our study population. The observation of ApoE allele distribution in this study shows variations in ApoE alleles in other populations [56, 58]. This distribution may significantly impact CVD risk and, subsequently, the development of cardiovascular disorders in our population.

Serum lipid parameters showed that ApoE*ε4 allele carriers have elevated TC, TG and LDL-C compared to ε2+ carriers and ε3/ε3. ApoE*ε4 has been shown to influence total cholesterol and LDL cholesterol even at lower body mass indices (BMIs) [59]. Other studies have shown that ApoE*ε4+ carriers in comparison to non-carriers have higher levels of TC, TG and LDL [60, 61], which is consistent with our findings. Another study has found that among HIV-positive patients who are ApoE*ε4 carriers, there is an elevation of TC, LDL-C, and TG which is associated with faster rates of cognitive decline [62].

A multiple regression analysis was performed to evaluate the independent ApoE allele carrier status predictors for CVD risk by adjusting conventional factors such as SBP, DBP, gender, age, smoking, BMI, and ApoE allele carrier status (ε2, ε3/ ε3, ε4). Classifications of atherogenic indices were based on reference limits and risk of CVD. After adjusting for the different variables, rs429358 CT/CC served as an independent significant risk factor for elevated TG (p = 0.025, OR = 0.28, 95%*CI* 0.09–0.85), LDL-C (p = 0.023, OR = 0.35, 95% *CI* 0.13–0.86) and AIP (p = 0.023, OR = 3.04, 95% CI 1.16–7.90). Studies conducted in Russia have confirmed associations between rs429358 genotypes and serum lipid parameters that pose a risk for cardiovascular diseases [63, 64].

ApoE*ε4 allele carrier status was significantly associated with TG (p = 0.015, OR = 0.20, 95% CI 0.05–0.73). ε4 allele has been found to be an independent predictor of coronary artery disease (CAD) (OR 2.32, 95%CI 1.17–4.61, p = 0.016) and type 2 diabetes (OR 2.04, 95%CI 1.07–3.86, p = 0.029) [65, 66]. ApoE*ε2+ was significantly associated (p = 0.030, OR = 0.24, 95% CI 0.06–0.87) with overweight and obesity (BMI>25.kg/m^2^) and Castelli risk index (p = 0.024, OR = 11.26, 95% CI .37–92.30) in females. The ApoE*ε2 isoform was found to be significantly associated with BMI and waist circumference in a multivariate model [67, 68]. Rs7412 CT/CC was significantly associated with BMI >25kg/m^2^ (p = 0.041, OR = 0.39, 95% CI 0.15–0.96) and Castelli risk index in females (p = 0.031, OR = 14.12, 95% CI 1.27–156.89). In contrast to our study, TT homozygous of rs7412 was significantly associated with BMI in a previous study in men [67].

Considering the influence of Apo E genetic variations on dyslipidemias, a 10-year risk analysis was undertaken using the QRISK-3 and Framingham BMI and cholesterol risk calculators. It was observed that 6–20% of malaria patients had a higher 10-year CVD risk using the three calculators. Malaria has been implicated in high blood pressure, where a link was established between malaria and high BP, which is a CVD risk factor [69]. In two previous metanalyses, potential links between malaria and cardiovascular diseases were observed, calling for further exploration in clinical studies [70, 71]. ApoE*ε4+ carriers stratified by ε3/ε4 and ε4/ε4 genotypes were at higher CVD risk. Several studies have elucidated the risk of carrying at least one copy of ε4 with cardiovascular risk which agrees with what was observed in our study [72, 73].

Accumulating evidence has shown that ApoE genotypes informs pre-symptomatic risk for a wide variety of diseases and is valuable for the diagnosis of type III dysbetalipoproteinemia and appears to impact the efficacy of certain drugs [74, 75]. Understanding interactions involving ApoE might yield potential for disease prevention in particular importance to those with a family history of dylipideamias.

### Study limitations

We acknowledge that the sample size in the various groups is not large enough. They are however representative. For comparative purposes, we did not use so-called healthy controls for comparative purposes but rather included controls that had higher lipid parameters for purpose of comparing representative genotypes in our population and disease status.

## Conclusion

In summary, ε2/ε2 genotypes are less represented in our population, whilst our study has shown that carrying ApoE*ε4 presents with higher serum levels of TC, TG and LDL-C and a higher 10-year risk of cardiovascular disease. Malaria patients seem to have a higher cardiovascular risk although the mechanism through which this occurs is yet to be elucidated. We suggest undertaking further studies to establish how this occurs.

## Supporting information

S1 Table ApoE *rs429358* variation and biochemical markers of atherogenic risks. (DOCX)

S2 Table ApoE *rs7412* variation and biochemical markers of atherogenic risks. (DOCX)

S3 Table ApoE status/genotype and biochemical markers of atherogenic risks. (DOCX)

S1 File (DOCX)

## References

[1] Feingold K. Introduction to Lipids and Lipoproteins. In: In: Feingold KR AB, Boyce A, et al., editors, editor. ENDOTEXT. South Dartmouth (MA): MDText.com, Inc.; 2000.

[2] Olson RE. Discovery of the Lipoproteins, Their Role in Fat Transport and Their Significance as Risk Factors. The Journal of Nutrition. 1998;128(2):439S–43S. doi: 10.1093/jn/128.2.439S 9478044

[3] Mahley RW. Apolipoprotein E: from cardiovascular disease to neurodegenerative disorders. J Mol Med (Berl). 2016;94(7):739–46. Epub 20160609. doi: 10.1007/s00109-016-1427-y ; PubMed Central PMCID: PMC4921111. 27277824 PMC4921111

[4] Satizabal CL, Samieri C, Davis-Plourde KL, Voetsch B, Aparicio HJ, Pase MP, et al. APOE and the Association of Fatty Acids With the Risk of Stroke, Coronary Heart Disease, and Mortality. Stroke. 2018;49(12):2822–9. doi: 10.1161/STROKEAHA.118.022132 ; PubMed Central PMCID: PMC6310220. 30571417 PMC6310220

[5] Liu S, Liu J, Weng R, Gu X, Zhong Z. Apolipoprotein E gene polymorphism and the risk of cardiovascular disease and type 2 diabetes. BMC Cardiovascular Disorders. 2019;19(1):213. doi: 10.1186/s12872-019-1194-0 31521122 PMC6744677

[6] Eichner JE, Dunn ST, Perveen G, Thompson DM, Stewart KE, Stroehla BC. Apolipoprotein E Polymorphism and Cardiovascular Disease: A HuGE Review. American Journal of Epidemiology. 2002;155(6):487–95. doi: 10.1093/aje/155.6.487 11882522

[7] Ginsberg HN, Packard CJ, Chapman MJ, Borén J, Aguilar-Salinas CA, Averna M, et al. Triglyceride-rich lipoproteins and their remnants: metabolic insights, role in atherosclerotic cardiovascular disease, and emerging therapeutic strategies—a consensus statement from the European Atherosclerosis Society. European Heart Journal. 2021;42(47):4791–806. doi: 10.1093/eurheartj/ehab551 34472586 PMC8670783

[8] Getz GS, Reardon CA. Apoprotein E as a lipid transport and signaling protein in the blood, liver, and artery wall. J Lipid Res. 2009;50 Suppl(Suppl):S156–61. Epub 20081118. doi: 10.1194/jlr.R800058-JLR200 ; PubMed Central PMCID: PMC2674757. 19018038 PMC2674757

[9] Rizzo G, Laganà AS. The Link between Homocysteine and Omega-3 Polyunsaturated Fatty Acid: Critical Appraisal and Future Directions. Biomolecules. 2020;10(2). Epub 20200202. doi: 10.3390/biom10020219 ; PubMed Central PMCID: PMC7072208. 32024302 PMC7072208

[10] Rizzo G, Laganà AS, Rapisarda AM, La Ferrera GM, Buscema M, Rossetti P, et al. Vitamin B12 among Vegetarians: Status, Assessment and Supplementation. Nutrients. 2016;8(12). Epub 20161129. doi: 10.3390/nu8120767 ; PubMed Central PMCID: PMC5188422. 27916823 PMC5188422

[11] Saleh RNM, West AL, Ostermann AI, Schebb NH, Calder PC, Minihane AM. APOE Genotype Modifies the Plasma Oxylipin Response to Omega-3 Polyunsaturated Fatty Acid Supplementation in Healthy Individuals. Frontiers in Nutrition. 2021;8. doi: 10.3389/fnut.2021.723813 34604280 PMC8484638

[12] Kern S, Mehlig K, Kern J, Zetterberg H, Thelle D, Skoog I, et al. The Distribution of Apolipoprotein E Genotype Over The Adult Lifespan and in Relation to Country of Birth. American Journal of Epidemiology. 2015;181(3):214–7. doi: 10.1093/aje/kwu442 25609095

[13] Hao L, Jia J, Xing Y, Han Y. APOE ε4 Allele Distribution and Association With Scores of Subjective Cognitive Decline Questionnaire 9 in a Large Chinese Memory Clinic Cohort. Front Neurosci. 2022;16:829031. Epub 20220603. doi: 10.3389/fnins.2022.829031 ; PubMed Central PMCID: PMC9204235. 35720695 PMC9204235

[14] González HM, Tarraf W, Jian X, Vásquez PM, Kaplan R, Thyagarajan B, et al. Apolipoprotein E genotypes among diverse middle-aged and older Latinos: Study of Latinos-Investigation of Neurocognitive Aging results (HCHS/SOL). Scientific Reports. 2018;8(1):17578. doi: 10.1038/s41598-018-35573-3 30546063 PMC6292877

[15] Acharya P, Garg M, Kumar P, Munjal A, Raja KD. Host-Parasite Interactions in Human Malaria: Clinical Implications of Basic Research. Front Microbiol. 2017;8:889. Epub 20170518. doi: 10.3389/fmicb.2017.00889 ; PubMed Central PMCID: PMC5435807. 28572796 PMC5435807

[16] Luckhart S, Pakpour N, Giulivi C. Host-pathogen interactions in malaria: cross-kingdom signaling and mitochondrial regulation. Curr Opin Immunol. 2015;36:73–9. Epub 20150723. doi: 10.1016/j.coi.2015.07.002 ; PubMed Central PMCID: PMC4593738. 26210301 PMC4593738

[17] Geisler CE, Hepler C, Higgins MR, Renquist BJ. Hepatic adaptations to maintain metabolic homeostasis in response to fasting and refeeding in mice. Nutrition & Metabolism. 2016;13(1):62. doi: 10.1186/s12986-016-0122-x 27708682 PMC5037643

[18] Liu M, Cao H, Hou Y, Sun G, Li D, Wang W. Liver Plays a Major Role in FGF-21 Mediated Glucose Homeostasis. Cellular Physiology and Biochemistry. 2018;45(4):1423–33. doi: 10.1159/000487568 29462809

[19] Kluck GEG, Wendt CHC, Imperio GEd, Araujo MFC, Atella TC, da Rocha, et al. Plasmodium Infection Induces Dyslipidemia and a Hepatic Lipogenic State in the Host through the Inhibition of the AMPK-ACC Pathway. Scientific Reports. 2019;9(1):14695. doi: 10.1038/s41598-019-51193-x 31604978 PMC6789167

[20] Mohanty S, Mishra SK, Das BS, Satpathy SK, Mohanty D, Patnaik JK, et al. Altered plasma lipid pattern in falciparum malaria. Ann Trop Med Parasitol. 1992;86(6):601–6. doi: 10.1080/00034983.1992.11812715 . 1304701

[21] Koch M, Cegla J, Jones B, Lu Y, Mallat Z, Blagborough A, et al. The effects of dyslipidaemia and cholesterol modulation on erythrocyte susceptibility to malaria parasite infection. bioRxiv. 2019:630251. doi: 10.1186/s12936-019-3016-3 31783858 PMC6884832

[22] Scaccabarozzi D, Deroost K, Lays N, Omodeo Salè F, Van den Steen PE, Taramelli D. Altered Lipid Composition of Surfactant and Lung Tissue in Murine Experimental Malaria-Associated Acute Respiratory Distress Syndrome. PLOS ONE. 2015;10(12):e0143195. doi: 10.1371/journal.pone.0143195 26624290 PMC4666673

[23] Visser BJ, Wieten RW, Nagel IM, Grobusch MP. Serum lipids and lipoproteins in malaria—a systematic review and meta-analysis. Malar J. 2013;12:442. Epub 20131207. doi: 10.1186/1475-2875-12-442 ; PubMed Central PMCID: PMC4029227. 24314058 PMC4029227

[24] Malvestutto CD, Aberg JA. Management of dyslipidemia in HIV-infected patients. Clin Lipidol. 2011;6(4):447–62. doi: 10.2217/clp.11.25 ; PubMed Central PMCID: PMC3249059. 22216062 PMC3249059

[25] Fiseha T, Alemu W, Dereje H, Tamir Z, Gebreweld A. Prevalence of dyslipidaemia among HIV-infected patients receiving combination antiretroviral therapy in North Shewa, Ethiopia. PLOS ONE. 2021;16(4):e0250328. doi: 10.1371/journal.pone.0250328 33905435 PMC8078799

[26] Maggi P, Di Biagio A, Rusconi S, Cicalini S, D’Abbraccio M, d’Ettorre G, et al. Cardiovascular risk and dyslipidemia among persons living with HIV: a review. BMC infectious diseases. 2017;17(1):551. doi: 10.1186/s12879-017-2626-z 28793863 PMC5550957

[27] Lakshminarasimhappa MC. Web-Based and Smart Mobile App for Data Collection: Kobo Toolbox / Kobo Collect. 2022. 2022;57(2):8. Epub 2022-02-06.

[28] Akpınar O, Bozkurt A, Acartürk E, Seydaoğlu G. A new index (CHOLINDEX) in detecting coronary artery disease risk. Anadolu Kardiyol Derg. 2013;13(4):315–9. Epub 20130326. doi: 10.5152/akd.2013.098 . 23531868

[29] Oguejiofor OC, Onwukwe CH, Odenigbo CU. Dyslipidemia in Nigeria: prevalence and pattern. Ann Afr Med. 2012;11(4):197–202. doi: 10.4103/1596-3519.102846 . 23103917

[30] Jahangiry L, Farhangi MA, Rezaei F. Framingham risk score for estimation of 10-years of cardiovascular diseases risk in patients with metabolic syndrome. Journal of Health, Population and Nutrition. 2017;36(1):36. doi: 10.1186/s41043-017-0114-0 29132438 PMC5682637

[31] Hippisley-Cox J, Coupland C, Brindle P. Development and validation of QRISK3 risk prediction algorithms to estimate future risk of cardiovascular disease: prospective cohort study. BMJ. 2017;357:j2099. doi: 10.1136/bmj.j2099 28536104 PMC5441081

[32] Zhu L, Singh M, Lele S, Sahakian L, Grossman J, Hahn B, et al. Assessing the validity of QRISK3 in predicting cardiovascular events in systemic lupus erythematosus. Lupus Science & Medicine. 2022;9(1):e000564. doi: 10.1136/lupus-2021-000564 35193947 PMC8867320

[33] Phillips MC. Apolipoprotein E isoforms and lipoprotein metabolism. IUBMB Life. 2014;66(9):616–23. doi: 10.1002/iub.1314 . 25328986

[34] Marais AD. Apolipoprotein E in lipoprotein metabolism, health and cardiovascular disease. Pathology. 2019;51(2):165–76. Epub 20181228. doi: 10.1016/j.pathol.2018.11.002 . 30598326

[35] Ogbera AO, Fasanmade OA, Chinenye S, Akinlade A. Characterization of lipid parameters in diabetes mellitus—a Nigerian report. Int Arch Med. 2009;2(1):19. Epub 20090720. doi: 10.1186/1755-7682-2-19 ; PubMed Central PMCID: PMC2734749. 19619328 PMC2734749

[36] Ogunleye OO, Ogundele SO, Akinyemi JO, Ogbera AO. Clustering of hypertension, diabetes mellitus and dyslipidemia in a Nigerian population: a cross sectional study. Afr J Med Med Sci. 2012;41(2):191–5. . 23185918

[37] Hou J, Deng Q, Guo X, Deng X, Zhong W, Zhong Z. Association between apolipoprotein E gene polymorphism and the risk of coronary artery disease in Hakka postmenopausal women in southern China. Lipids Health Dis. 2020;19(1):139. Epub 20200616. doi: 10.1186/s12944-020-01323-6 ; PubMed Central PMCID: PMC7298959. 32546237 PMC7298959

[38] Rajan KB, Barnes LL, Wilson RS, McAninch EA, Weuve J, Sighoko D, et al. Racial Differences in the Association Between Apolipoprotein E Risk Alleles and Overall and Total Cardiovascular Mortality Over 18 Years. J Am Geriatr Soc. 2017;65(11):2425–30. Epub 20170912. doi: 10.1111/jgs.15059 ; PubMed Central PMCID: PMC6201232. 28898389 PMC6201232

[39] Marron MM, Moore SC, Wendell SG, Boudreau RM, Miljkovic I, Sekikawa A, et al. Using lipid profiling to better characterize metabolic differences in apolipoprotein E (APOE) genotype among community-dwelling older Black men. Geroscience. 2022;44(2):1083–94. Epub 20210515. doi: 10.1007/s11357-021-00382-6 ; PubMed Central PMCID: PMC9135949. 33991295 PMC9135949

[40] González-Domínguez R, Castellano-Escuder P, Lefèvre-Arbogast S, Low DY, Du Preez A, Ruigrok SR, et al. Apolipoprotein E and sex modulate fatty acid metabolism in a prospective observational study of cognitive decline. Alzheimer’s Research & Therapy. 2022;14(1):1. doi: 10.1186/s13195-021-00948-8 34980257 PMC8725342

[41] Sun Y, Wei R, Yan D, Xu F, Zhang X, Zhang B, et al. Association between APOE polymorphism and metabolic syndrome in Uyghur ethnic men. BMJ Open. 2016;6(1):e010049. doi: 10.1136/bmjopen-2015-010049 26739741 PMC4716259

[42] Krishna AP, Chandrika, Kumar S, Acharya M, Patil SL. Variation in common lipid parameters in malaria infected patients. Indian J Physiol Pharmacol. 2009;53(3):271–4. . 20329375

[43] Visser BJ, de Vries SG, Vingerling R, Gritter M, Kroon D, Aguilar LC, et al. Serum Lipids and Lipoproteins During Uncomplicated Malaria: A Cohort Study in Lambaréné, Gabon. Am J Trop Med Hyg. 2017;96(5):1205–14. doi: 10.4269/ajtmh.16-0721 ; PubMed Central PMCID: PMC5417218. 28500816 PMC5417218

[44] Babaliche P, Gubba P. Variation in common serum lipid parameters in patients with malaria: A 1-year cross-sectional study. Journal of Current Research in Scientific Medicine. 2019;5(1):39–43. doi: 10.4103/jcrsm.jcrsm_1_19

[45] Neves FA, Ventura AM, Filho MG, Libonati RM. Lipid parameters in a hyperendemic area for malaria. Lipids Health Dis. 2013;12:162. Epub 20131101. doi: 10.1186/1476-511X-12-162 ; PubMed Central PMCID: PMC3826618. 24180363 PMC3826618

[46] Khovidhunkit W, Kim MS, Memon RA, Shigenaga JK, Moser AH, Feingold KR, et al. Effects of infection and inflammation on lipid and lipoprotein metabolism: mechanisms and consequences to the host. J Lipid Res. 2004;45(7):1169–96. Epub 20040421. doi: 10.1194/jlr.R300019-JLR200 . 15102878

[47] Badiaga S, Barrau K, Parola P, Brouqui P, Delmont J. Contribution of nonspecific laboratory test to the diagnosis of malaria in febrile travelers returning from endemic areas: value of hypocholesterolemia. J Travel Med. 2002;9(3):117–21. doi: 10.2310/7060.2002.23842 . 12088575

[48] Adewole OO, Eze S, Betiku Y, Anteyi E, Wada I, Ajuwon Z, et al. Lipid profile in HIV/AIDS patients in Nigeria. Afr Health Sci. 2010;10(2):144–9. ; PubMed Central PMCID: PMC2956300. 21326966 PMC2956300

[49] Bekolo CE, Nguena MB, Ewane L, Bekoule PS, Kollo B. The lipid profile of HIV-infected patients receiving antiretroviral therapy in a rural Cameroonian population. BMC Public Health. 2014;14(1):236. doi: 10.1186/1471-2458-14-236 24606888 PMC3973972

[50] Singh J, Verma M, Ghalaut PS, Verma R, Soni A, Ghalaut VS. Alteration in Lipid Profile in Treatment-Naive HIV-Infected Patients and Changes Following HAART Initiation in Haryana2014.

[51] Melzi S, Carenzi L, Cossu MV, Passerini S, Capetti A, Rizzardini G. Lipid Metabolism and Cardiovascular Risk in HIV-1 Infection and HAART: Present and Future Problems. Cholesterol. 2010;2010:271504. doi: 10.1155/2010/271504 21490912 PMC3065849

[52] Oka F, Naito T, Oike M, Imai R, Saita M, Inui A, et al. Correlation between HIV disease and lipid metabolism in antiretroviral-naïve HIV-infected patients in Japan. Journal of Infection and Chemotherapy. 2012;18(1):17–21. doi: 10.1007/s10156-011-0275-5 21735099 PMC3278606

[53] Peltenburg NC, Schoeman JC, Hou J, Mora F, Harms AC, Lowe SH, et al. Persistent metabolic changes in HIV-infected patients during the first year of combination antiretroviral therapy. Scientific Reports. 2018;8(1):16947. doi: 10.1038/s41598-018-35271-0 30446683 PMC6240055

[54] Atis O, Sahin S, Ceyhan K, Ozyurt H, Akbas A, Benli I. The Distribution of Apolipoprotein E Gene Polymorphism and Apolipoprotein E Levels among Coronary Artery Patients Compared to Controls. The Eurasian journal of medicine. 2016;48(2):90–4. doi: 10.5152/eurasianjmed.2015.25 ; PubMed Central PMCID: PMC4970561. 27551170 PMC4970561

[55] Egert S, Rimbach G, Huebbe P. ApoE genotype: from geographic distribution to function and responsiveness to dietary factors. Proceedings of the Nutrition Society. 2012;71(3):410–24. Epub 2012/05/08. doi: 10.1017/S0029665112000249 22564824

[56] Touré M, Diouf NN, Thiam S, Diop JP, Coly MS, Mbengue A, et al. Frequencies and Distribution of APOE Gene Polymorphisms and Its Association With Lipid Parameters in the Senegalese Population. Cureus. 2022;14(4):e24063. Epub 20220412. doi: 10.7759/cureus.24063 ; PubMed Central PMCID: PMC9097468. 35573533 PMC9097468

[57] Khabour OF, Abdelhalim ES. Distribution of APOE gene variations in the Jordanian population: Association with longevity. Journal of King Saud University—Science. 2020;32(1):518–22. doi: 10.1016/j.jksus.2018.08.004

[58] Singh PP, Singh M, Mastana SS. APOE distribution in world populations with new data from India and the UK. Ann Hum Biol. 2006;33(3):279–308. doi: 10.1080/03014460600594513 . 17092867

[59] Garcia AR, Finch C, Gatz M, Kraft T, Eid Rodriguez D, Cummings D, et al. APOE4 is associated with elevated blood lipids and lower levels of innate immune biomarkers in a tropical Amerindian subsistence population. Elife. 2021;10. Epub 20210929. doi: 10.7554/eLife.68231 ; PubMed Central PMCID: PMC8480980. 34586066 PMC8480980

[60] Yasuno F, Asada T. Effect of plasma lipids and APOE genotype on cognitive decline. Dialogues in Clinical Neuroscience. 2013;15(1):120–6. doi: 10.31887/DCNS.2013.15.1/fyasuno 23576895 PMC3622465

[61] Gan C, Zhang Y, Liang F, Guo X, Zhong Z. Effects of APOE gene ε4 allele on serum lipid profiles and risk of cardiovascular disease and tumorigenesis in southern Chinese population. World J Surg Oncol. 2022;20(1):280. Epub 20220903. doi: 10.1186/s12957-022-02748-2 ; PubMed Central PMCID: PMC9440530. 36057714 PMC9440530

[62] Mukerji SS, Locascio JJ, Misra V, Lorenz DR, Holman A, Dutta A, et al. Lipid Profiles and APOE4 Allele Impact Midlife Cognitive Decline in HIV-Infected Men on Antiretroviral Therapy. Clin Infect Dis. 2016;63(8):1130–9. Epub 20160722. doi: 10.1093/cid/ciw495 ; PubMed Central PMCID: PMC5036920. 27448678 PMC5036920

[63] Semaev S, Shakhtshneider E, Shcherbakova L, Ivanoshchuk D, Orlov P, Malyutina S, et al. Associations of APOE Gene Variants rs429358 and rs7412 with Parameters of the Blood Lipid Profile and the Risk of Myocardial Infarction and Death in a White Population of Western Siberia. Curr Issues Mol Biol. 2022;44(4):1713–24. Epub 20220413. doi: 10.3390/cimb44040118 ; PubMed Central PMCID: PMC9164079. 35723376 PMC9164079

[64] Wu H, Huang Q, Yu Z, Wu H, Zhong Z. The SNPs rs429358 and rs7412 of APOE gene are association with cerebral infarction but not SNPs rs2306283 and rs4149056 of SLCO1B1 gene in southern Chinese Hakka population. Lipids in Health and Disease. 2020;19(1):202. doi: 10.1186/s12944-020-01379-4 32891149 PMC7487494

[65] Chaudhary R, Likidlilid A, Peerapatdit T, Tresukosol D, Srisuma S, Ratanamaneechat S, et al. Apolipoprotein E gene polymorphism: effects on plasma lipids and risk of type 2 diabetes and coronary artery disease. Cardiovasc Diabetol. 2012;11:36. Epub 20120423. doi: 10.1186/1475-2840-11-36 ; PubMed Central PMCID: PMC3372424. 22520940 PMC3372424

[66] Wu L, Zhang Y, Zhao H, Rong G, Huang P, Wang F, et al. Dissecting the Association of Apolipoprotein E Gene Polymorphisms With Type 2 Diabetes Mellitus and Coronary Artery Disease. Front Endocrinol (Lausanne). 2022;13:838547. Epub 20220208. doi: 10.3389/fendo.2022.838547 ; PubMed Central PMCID: PMC8861372. 35211094 PMC8861372

[67] Tejedor MT, Garcia-Sobreviela MP, Ledesma M, Arbones-Mainar JM. The apolipoprotein E polymorphism rs7412 associates with body fatness independently of plasma lipids in middle aged men. PLoS One. 2014;9(9):e108605. Epub 20140930. doi: 10.1371/journal.pone.0108605 ; PubMed Central PMCID: PMC4182517. 25268647 PMC4182517

[68] Zeljko HM, Škarić-Jurić T, Narančić NS, Tomas Ž, Barešić A, Salihović MP, et al. E2 allele of the Apolipoprotein E gene polymorphism is predictive for obesity status in Roma minority population of Croatia. Lipids in Health and Disease. 2011;10(1):9. doi: 10.1186/1476-511X-10-9 21244662 PMC3025844

[69] Etyang AO, Kapesa S, Odipo E, Bauni E, Kyobutungi C, Abdalla M, et al. Effect of Previous Exposure to Malaria on Blood Pressure in Kilifi, Kenya: A Mendelian Randomization Study. Journal of the American Heart Association. 2019;8(6):e011771. doi: 10.1161/JAHA.118.011771 30879408 PMC6475058

[70] Holm A, Gomes LC, Biering-Soerensen T, Silvestre O, Brainin P. Malaria and cardiovascular disease: a systematic review. European Heart Journal. 2020;41(Supplement_2). doi: 10.1093/ehjci/ehaa946.2817

[71] Holm AE, Gomes LC, Marinho CRF, Silvestre OM, Vestergaard LS, Biering-Sørensen T, et al. Prevalence of Cardiovascular Complications in Malaria: A Systematic Review and Meta-Analysis. Am J Trop Med Hyg. 2021;104(5):1643–50. Epub 20210315. doi: 10.4269/ajtmh.20-1414 ; PubMed Central PMCID: PMC8103436. 33724926 PMC8103436

[72] Tsiknia AA, Reas E, Bangen KJ, Sundermann EE, McEvoy L, Brewer JB, et al. Sex and APOE ɛ4 modify the effect of cardiovascular risk on tau in cognitively normal older adults. Brain Communications. 2022;4(1). doi: 10.1093/braincomms/fcac035 35233525 PMC8882003

[73] Chen W, Jin F, Cao G, Mei R, Wang Y, Long P, et al. ApoE4 May be a Promising Target for Treatment of Coronary Heart Disease and Alzheimer’s Disease. Curr Drug Targets. 2018;19(9):1038–44. doi: 10.2174/1389450119666180406112050 . 29623835

[74] Smelt AH, de Beer F. Apolipoprotein E and familial dysbetalipoproteinemia: clinical, biochemical, and genetic aspects. Semin Vasc Med. 2004;4(3):249–57. doi: 10.1055/s-2004-861492 . 15630634

[75] Murphy MS O’Brien T. CHAPTER 23—DYSLIPIDEMIAS. In: Waldman SA, Terzic A, Egan LJ, Elghozi J-L, Jahangir A, Kane GC, et al., editors. Pharmacology and Therapeutics. Philadelphia: W.B. Saunders; 2009. p. 303–20.

