# Supplementary material for: Apolipoprotein E genetic variation, atherogenic index and cardiovascular disease risk assessment in an African population: An analysis of HIV and malaria patients in Ghana
Source: PLoS One. 2023 May 3;18(5):e0284697. doi: 10.1371/journal.pone.0284697 (PMC10155972; doi:10.1371/journal.pone.0284697)
Supplement: S1 Table — (DOCX) [file pone.0284697.s001.docx]

**S1 Table ApoE *rs429358* variation and biochemical markers of d atherogenic risks**

|  | **Malaria (n=76)** | | | | **HIV (n=33)** | | | | **Malaria-HIV (n=21)** | | | | **CONTROLS (n=31)** | | | |
| --- | --- | --- | --- | --- | --- | --- | --- | --- | --- | --- | --- | --- | --- | --- | --- | --- |
|  | ***APOE rs429358*** | | | | ***APOE rs429358*** | | | | ***APOE rs429358*** | | | | ***APOE rs429358*** | | | |
|  | T/T | C/T | C/C | p-value | T/T | C/T | C/C | p-value | T/T | C/T | C/C | p-value | T/T | C/T | C/C | p-value |
| TC (mmol/L) | 3.71 ± 1.15 | 4.65 ± 2.24 | 3.72 ± 0.79 | 0.105 | 3.78 ± 1.00 | 4.62 ± 1.09 | 4.53 ± 0.10 | 0.032* | 3.06 ± 1.11 | 4.19 ± 0.61 | - | 0.000* | 5.62 ± 1.34 | 5.09 ± 1.92 | 6.07 ± 0.64 | 0.493 |
| TG | 1.17 ± 0.55 | 1.60 ± 0.74 | 1.81 ± 1.29 | 0.020* | 1.37 ± 0.93 | 1.53 ± 0.61 | 0.83 ± 0.00 | 0.553 | 1.61 ± 1.22 | 0.89 ± 0.20 | - | 0.123 | 1.37 ± 0.91 | 1.82 ± 0.82 | 2.17 ± 2.21 | 0.353 |
| HDL-C | 1.18 ± 0.72 | 1.11 ± 0.67 | 0.68 ± 0.23 | 0.245 | 1.07 ± 0.54 | 1.04 ± 0.35 | 1.03 ± 0.00 | 0.983 | 1.29 ± 1.11 | 1.61 ± 1.35 |  | 0.075 | 1.59 ± 0.58 | 1.37 ± 0.32 | 2.12 ± 1.46 | 0.225 |
| LDL-C | 1.94 ± 0.93 | 2.80 ± 2.08 | 2.21 ± 1.00 | 0.083 | 2.08 ± 0.78 | 2.89 ± 0.69 | 3.12 ± 0.00 | 0.013* | 1.031 ± 1.89 | 2.18 ± 0.83 | - | 0.316 | 3.40 ± 1.11 | 3.27 ± 1.28 | 2.97 ± 0.68 | 0.739 |
| Non-HDL-C | 2.52 ± 0.98 | 3.53 ± 2.05 | 3.04 ± 0.69 | 0.029* | 2.70 ± 0.74 | 3.58 ± 0.85 | 3.50 ± 0.01 | 0.014* | 4.96 ± 4.11 | 3.78 ± 2.80 | - | 0.074 | 3.91 ± 1.41 | 4.73 ± 1.28 | 3.85 ± 1.89 | 0.426 |
| Chol/HDL ratio | 4.04 ± 2.95 | 4.82 ± 2.12 | 3.42 ± 1.84 | 0.574 | 4.43 ± 3.13 | 4.59 ± 0.85 | 4.3 ± 0.00 | 0.986 | 4.08 ± 2.79 | 3.79 ± 2.81 | - | 0.164 | 3.91 ± 1.01 | 3.83 ± 1.61 | 3.84 ± 1.88 | 0.991 |
