# Supplementary material for: Apolipoprotein E genetic variation, atherogenic index and cardiovascular disease risk assessment in an African population: An analysis of HIV and malaria patients in Ghana
Source: PLoS One. 2023 May 3;18(5):e0284697. doi: 10.1371/journal.pone.0284697 (PMC10155972; doi:10.1371/journal.pone.0284697)
Supplement: S2 Table — (DOCX) [file pone.0284697.s002.docx]

**S2 Table ApoE *rs7412* variation and biochemical markers of atherogenic risks**

|  | **Malaria (n=76)** | | | | **HIV (n=33)** | | | | **Malaria-HIV (n=21)** | | | | **CONTROLS (n=31)** | | | |
| --- | --- | --- | --- | --- | --- | --- | --- | --- | --- | --- | --- | --- | --- | --- | --- | --- |
|  | ***APOE rs7412*** | | |  | ***APOE rs7412*** | | |  | ***APOE rs7412*** | | |  | ***APOE rs7412*** | | |  |
|  | C/C | C/T | T/T | p-value | C/C | C/T | T/T | p-value | C/C | C/T | T/T | p-value | C/C | C/T | T/T |  |
| TC (mmol/L) | 4.01 ± 1.55 | 3.52 ± 0.82 | - | 0.016* | 4.16 ± 0.85 | 4.13 ± 1.37 | 3.45 ± 0.83 | 0.010* | 3.39 ± 1.17 | 2.52 ± 0.22 | - | 0.002* | 5.50 ± 1.37 | 7.70 ± 0.10 | - | 0.001* |
| TG | 1.40 ± 0.76 | 1.06 ± 0.47 | - | 0.001* | 1.21 ± 0.61 | 1.49 ± 0.89 | 2.04 ± 1.56 | 0.085 | 1.7 ± 1.13 | 0.44 ± 0.04 | - | 0.001* | 1.62 ± 1.19 | 1.11 ± 0.20 | - | 0.059 |
| HDL-C | 1.13 ± 0.77 | 1.09 ± 0.44 | - | 0.695 | 0.95 ± 0.27 | 1.24 ± 0.63 | 0.87 ± 0.49 | 0.007* | 1.37 ± 1.18 | 1.24 ± 0.69 | - | 0.665 | 1.62 ± 0.77 | 1.82 ± 0.10 | - | 0.245 |
| LDL-C | 2.19 ± 1.37 | 1.94 ± 0.72 | - | 0.161 | 2.66 ± 0.62 | 2.21 ± 1.02 | 1.65 ± 0.38 | 0.001* | 1.25 ± 0.97 | 1.07 ± 0.45 | - | 0.445 | 3.23 ± 1.02 | 5.37 ± 0.05 | - | 0.001* |
| Non-HDL-C | 2.86 ± 1.36 | 2.42 ± 0.87 | - | 0.019* | 3.21 ± 0.74 | 2.89 ± 1.05 | 2.58 ± 0.33 | 0.005* | 5.25 ± 0.04 | 2.34 ± 1.13 | - | 0.001 | 4.08 ± 1.48 | 4.23 ± 0.22 | - | 0.648 |
| Chol/HDL ratio | 4.61 ± 3.10 | 3.66 ± 1.50 | - | 0.017* | 4.53 ± 0.94 | 4.45 ± 0.87 | 4.40 ± 1.55 | 0.900 | 4.37 ± 2.79 | 2.35 ± 1.13 | - | 0.004* | 3.86 ± 1.50 | 4.23 ± 0.31 | - | 0.275 |
