# Supplementary material for: Apolipoprotein E genetic variation, atherogenic index and cardiovascular disease risk assessment in an African population: An analysis of HIV and malaria patients in Ghana
Source: PLoS One. 2023 May 3;18(5):e0284697. doi: 10.1371/journal.pone.0284697 (PMC10155972; doi:10.1371/journal.pone.0284697)
Supplement: S3 Table — (DOCX) [file pone.0284697.s003.docx]

**S3 Table ApoE status/genotype and Biochemical markers of atherogenic risks**

|  | **Malaria (n=76)** | | | | | | | **HIV (n=33)** | | | | | | |
| --- | --- | --- | --- | --- | --- | --- | --- | --- | --- | --- | --- | --- | --- | --- |
|  | ε2/ε4 | ε3/ε3 | ε2/ε3 | ε3/ε4 | ε4/ε4 | ε2/ε2 | p-value | ε2/ε4 | ε3/ε3 | ε2/ε3 | ε3/ε4 | ε4/ε4 | ε2/ε2 | p-value |
| TC (mmol/L) | 4.26 + 0.48 | 3.87 + 1.27 | 3.35 + 0.78 | 4.79 + 2.65 | 3.86 + 0.79 | 3.45 + 0.83 | <0.0001* | 5.41 + 1.09 | 3.94 + 0.79 | 3.77 + 1.26 | 4.30 + 1.04 | 4.53 + 0.10 | 3.45 + 0.83 | 0.103 |
| TG | 1.31 + 0.47 | 1.24 + 0.57 | 1.21 + 0.94 | 1.71 + 0.82 | 1.32 + 0.54 |  | 0.259 | 1.40 + 0.08 | 0.91 + 0.27 | 1.51 + 1.03 | 1.58 + 0.74 | 0.83 + 0.10 | 2.04 + 1.56 | 0.077 |
| HDL-C | 1.28 + 0.47 | 1.25 + 0.82 | 1.03 + 0.45 | 1.06 + 0.75 | 0.74 + 0.25 |  | 0.009* | 1.40 + 0.60 | 0.99 + 0.41 | 1.20 + 0.67 | 0.89 + 0.12 | 1.03 + 0.01 | 0.87 + 0.49 | 0.509 |
| LDL-C | 2.38 + 0.17 | 1.99 + 1.0 | 1.77 + 0.81 | 2.96 + 0.47 | 2.55 + 0.61 |  | 0.007* | 3.38 + 0.46 | 2.53 + 0.62 | 1.87 + 0.87 | 2.69 + 0.71 | 3.12 + 0.02 | 1.65 + 0.38 | 0.006* |
| Non-HDL-C | 2.98 + 0.04 | 2.62 + 1.01 | 2.32 + 0.88 | 3.74 + 2.42 | 3.15 + 0.71 |  | 0.019* | 4.01 + 0.50 | 2.94 + 0.54 | 2.57 + 0.94 | 3.41 + 0.95 | 3.5 + 0.01 | 2.56 + 0.33 | 0.033* |
| Chol/HDL ratio | 3.54 + 0.94 | 4.20 + 0.41 | 3.80 + 1.62 | 5.29 + 2.29 | 5.90 + 2.06 |  | 0.0001* | 4.08 + 0.97 | 4.28 + 1.18 | 4.57 + 0.45 | 4.79 + 0.81 | 4.40 + 0.20 | 4.40 + 1.55 | 0.597 |

|  | **Malaria-HIV (n=33)** | | | | | | | **Control (n=31)** | | | | | | |
| --- | --- | --- | --- | --- | --- | --- | --- | --- | --- | --- | --- | --- | --- | --- |
|  | ε2/ε4 | ε3/ε3 | ε2/ε3 | ε3/ε4 | ε4/ε4 | ε2/ε2 | p-value | ε2/ε4 | ε3/ε3 | ε2/ε3 | ε3/ε4 | ε4/ε4 | ε2/ε2 | p-value |
| TC (mmol/L) |  | 3.19 + 1.21 | 2.52 + 0.22 | 4.19 + 0.61 | 4.44 ± 0.88 | - | 0.158 | - | 5.50 + 1.28 | 7.70 + 0.03 | 5.09 + 1.92 | 6.07 + 0.64 | - | 0.293 |
| TG |  | 1.90 + 1.19 | 0.44 + 0.04 | 0.89 + 0.21 | 2.23 ± 0.76 |  | 0.134 |  | 1.39 + 0.94 | 1.11 + 0.01 | 1.82 + 0.82 | 2.17 + 0.43 |  | 0.275 |
| HDL-C |  | 1.31 + 0.23 | 1.24 + 0.69 | 1.61 + 1.35 | 1.69 ± 0.66 |  | 0.752 |  | 1.58 + 0.60 | 1.82 + 0.10 | 1.37 + 0.33 | 2.12 + 1.47 |  | 0.397 |
| LDL-C |  | 2.14 + 1.02 | 1.07 + 0.45 | 2.18 + 0.83 | 2.45 ± 0.89 |  | 0.474 |  | 3.29 + 1.03 | 5.37 + 0.10 | 3.27 + 1.28 | 2.97 + 0.68 |  | 0.246 |
| Non-HDL-C |  | 5.62 + 0.37 | 2.34 + 1.13 | 3.78 + 0.80 | 5.87 ± 0.76 |  | 0.001* |  | 3.89 + 1.45 | 4.23 + 0.1 | 4.73 + 1.29 | 3.85 + 1.89 |  | 0.631 |
| Chol/HDL ratio |  | 4.52 + 2.96 | 2.35 + 1.13 | 3.79 + 2.81 | 2.63 ± 0.33 |  | 0.648 |  | 3.80 + 1.41 | 4.20 + 0.01 | 3.83 + 1.61 | 3.81 + 1.80 |  | 0.996 |
