# Supplementary material for: Apolipoprotein E genetic variation, atherogenic index and cardiovascular disease risk assessment in an African population: An analysis of HIV and malaria patients in Ghana
Source: PLoS One. 2023 May 3;18(5):e0284697. doi: 10.1371/journal.pone.0284697 (PMC10155972; doi:10.1371/journal.pone.0284697)
Supplement: S1 File — (DOCX) [file pone.0284697.s004.docx]

**S4 Supporting Information – Data availability statement**

Data can however be made available to interested researchers upon request following approval by the relevant institutional review boards. Request can be directed to the PHARMABIOME project (<http://pgmg-lab.com/pharmabiome-project>). Data sharing is in accordance with informed consent after an approval by the Cape Coast Teaching Hospital Ethical Review Board (CCTHERC) and University of Cape Coast Institutional Review Board (UCCIRB).
